# Causal relationship between Women’s reproductive traits and *postpartum* depression: a multivariate mendelian randomization analysis

**DOI:** 10.3389/fgene.2024.1434762

**Published:** 2024-10-11

**Authors:** Zhen Kang, Qingming Wu, Jianan Cao, Mohao Zhu, Zhaoling You, Dandan Li, Weiai Liu

**Affiliations:** ^1^ The Second Affiliated Hospital of Hunan University of Chinese Medicine, Changsha, China; ^2^ The First Affiliated Hospital of Hunan University of Chinese Medicine, Changsha, China

**Keywords:** women’s reproductive traits, *postpartum* depression, Mendelian randomization, GWAS, causal relationship

## Abstract

**Purpose:**

The relationship between women’s reproductive traits and *postpartum* depression (PPD) has not been clarified. We reveal the association between genetically predicted modifiable women’s reproductive traits and PPD using two-sample Mendelian randomization (MR).

**Methods:**

We used genome-wide association studies (GWASs) to obtain instrumental variables (IVs) of 9 women’s reproductive traits. Univariate and multivariate MR analyses were used to examine the association between traits and the risk of PPD (13,657 cases and 236,178 controls). The primary causal effect assessment employed the IVW method. Heterogeneity was assessed using Cochran’s Q test. Multiple horizontal effects were assessed using the MR-PRESSO and MR-Egger intercept. Leave-one-out and LASSO regression analyses were used to check the robustness of the UVMR and MVMR results, respectively.

**Results:**

In the UVMR result, genetic prediction showed that age at first sexual intercourse (AFS) (OR = 0.474, 95% CI 0.396–0.567; *p* = 4.6 × 10–16), age at first birth (AFB) (OR = 0.865, 95% CI 0.805–0.930; *p* = 8.02 × 10^−5^), and age at last live birth (ALLB) (OR = 0.296, 95% CI 0.138–0.636; *p* = 0.002) were significantly inversely associated with PPD, while a higher lifetime number of sexual partners (LNSP) (OR = 1.431, 95% CI 1.009–2.031; *p* = 0.045) and a greater number of spontaneous miscarriages (OR = 1.519, 95% CI 1.021–2.262; *p* = 0.039) are suggested to be associated with an increased risk of PPD. In the MVMR result, only AFB (OR = 0.804, 95% CI 0.661–0.978; *p* = 0.029) retained a direct causative relationship with PPD.

**Conclusion:**

The study indicates that AFB is a significant risk factor for PPD. Furthermore, the likelihood of developing PPD appears to decrease with increasing gestational age at the time of the first childbirth.

## 1 Introduction

*Postpartum* depression (PPD) is a frequent psychiatric illness during the puerperium that has a substantial negative impact on the mother’s life and the development of the child (O’Hara and McCabe, 2013), with a global prevalence of 5%–35% (Herba et al., 2016). Infants of moms with *postpartum* PPD have a variety of negative outcomes compared to infants of mothers without PPD. These include less self-regulation, increased symptoms of stress (Borairi et al., 2024), and more negative mother-infant relationships (Barnes and Theule, 2019). PPD may also lead to adolescent psychological and medical issues (Pearlstein et al., 2009). Furthermore, moms with PPD are less likely to start or continue breastfeeding (Ip et al., 2007). Suicide following PPD is the primary cause of maternal mortality (Lindahl et al., 2005; Johannsen et al., 2016). PPD is commonly defined as a *postpartum* episode of major depressive disorder (MDD) (American, 2013) and is also a risk factor or marker for bipolar disorder (Dudek et al., 2014). However, the time window for its diagnosis is controversial, as it may present with depressive symptoms during the current pregnancy, previous pregnancies, or the *postpartum* period (Yim et al., 2015). The complexity of the diagnosis often leads to its frequent clinical neglect and untimely intervention (O’Hara, 2009). Awareness of the significance of *postpartum* depression, coupled with proactive prevention, can reduce or eliminate the occurrence of depressive symptoms and have a long-term impact on the development of the offspring (Sockol et al., 2013; Brummelte and Galea, 2016).

There is a strong association between reproductive traits and PPD. According to our search (Ni et al., 2019; Yu et al., 2023; Brito et al., 2023), women’s reproductive traits include age at first sexual intercourse (AFS), age at menarche (AMC), age at first birth (AFB), age at menopause (AMP), lifetime number of sexual partners (LNSP), age at last live birth (ALLB), number of live births (NLB), number of stillbirths and number of spontaneous miscarriages. Some women’s reproductive traits are associated with depressive symptoms: the younger the AFS and AMP (Gonçalves et al., 2017; Georgakis et al., 2016) and the later the AMC (Kim et al., 2023), the more likely they are to develop depression. AFB was positively correlated with depression at a younger (before 20) or older (after 30) age, which may be related to age-related physical decrements and health issues (Carlson, 2011). A connection between depression and LNSP has been found in a cohort study with a predominantly African American population, but more research is required (Rubin et al., 2009). The number of miscarriages is considered an independent risk factor for PPD (Lin et al., 2022). There is limited evidence that AALB and NLB are linked to depression. Additionally, it remains uncertain whether stillbirth contributes to the development of depression during the *postpartum* period (Hennegan et al., 2015; Huberty et al., 2014). With participants from Europe, Asia, Africa, and other regions of the world, these studies suffer from a certain degree of racial heterogeneity. Inconsistency in research methods leads to difficulty in synthesizing the relationship between evaluation and depression. There are some scattered studies on the association between female reproductive characteristics and the risk of developing PPD, but there is insufficient comprehensive generalization and analysis.

The ethical constraints of observational research and the lengthy duration of epidemiologic cohort studies, both of which are costly to execute and subject to inescapable confounding factors, make it challenging to express the risk of women’s reproductive traits for PPD. Mendelian randomization (MR) studies depend on the Mendelian genetics principle of random assignment, and by using genetic variants from genome-wide association studies (GWASs) as instrumental variables (IVs), biases in clinical and experimental studies can be eliminated, and reverse causality bias can be avoided by excluding the interference of confounding factors (Holmes et al., 2017). MR studies more accurately identify potential causal relationships between exposure or risk variables and outcomes compared to randomized control trials (RCTs) (Sekula et al., 2016). With the current study, we conducted a two-sample multivariate Mendelian randomization analysis to examine the causal relationships between 9 women’s reproductive traits and PPD with the expectation that it may offer meaningful suggestions for clinical treatment and prevention.

## 2 Materials and methods

### 2.1 Study design

**FIGURE 1:**
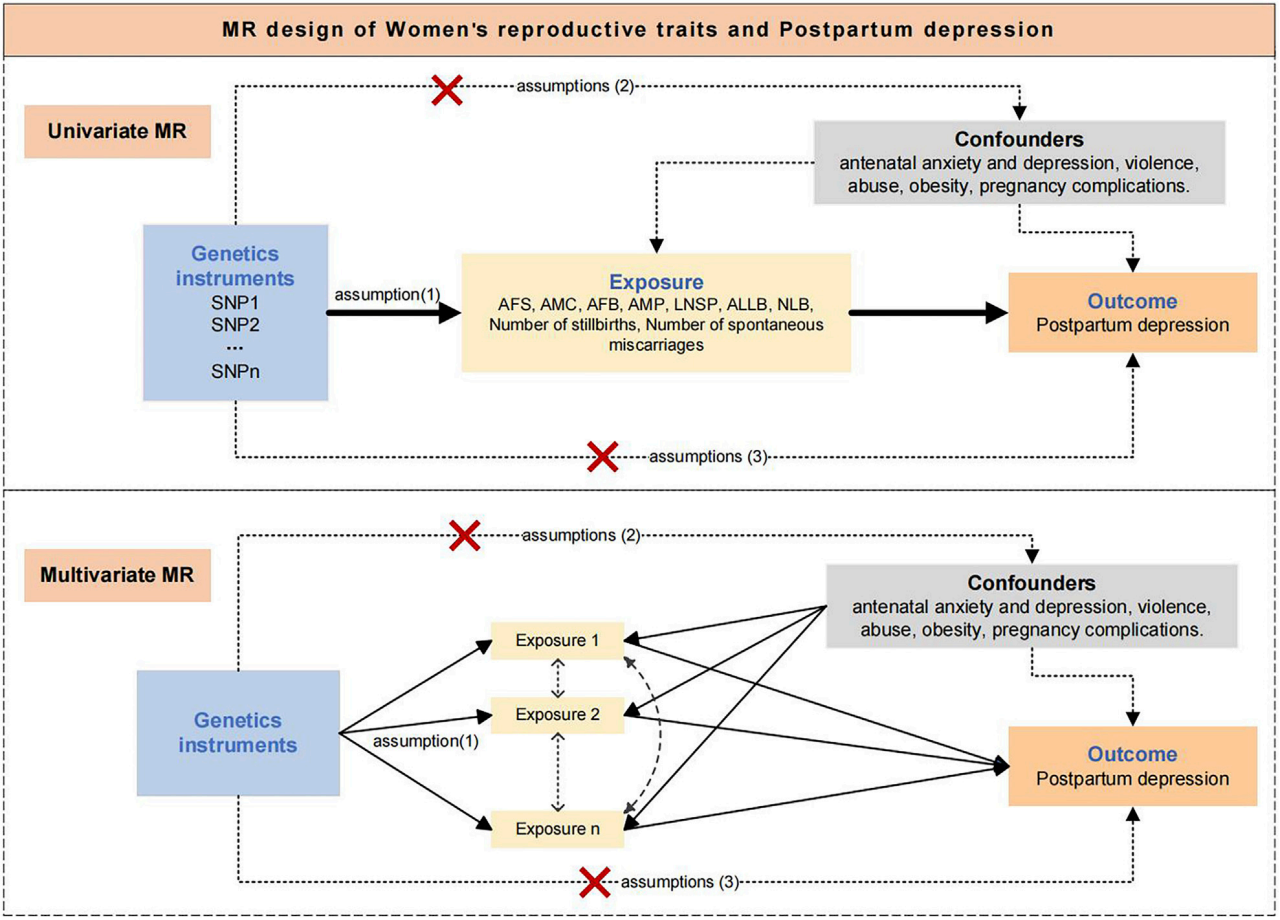
Assumptions and study design of the MR study of the associations between women's reproductive traits and postpartum depression. AFS, age at first sexual intercourse; AMC, age at menarche; AFB, age at first birth; AMP, age at menopause, LNSP, lifetime number of sexual partners; ALLB, age at last live birth; NLB, number of live births; PPD, Postpartum depression.

The design of this study is shown in Figure 1. We used Two-sample Univariable MR (UVMR) and Multivariable MR (MVMR) to explore the influence of 9 women’s reproductive traits on PPD. UVMR involved 3 core assumptions (Smith and Ebrahim, 2004). 1) single nucleotide polymorphisms (SNPs) are strongly associated with women’s reproductive traits; 2) SNPs affect *postpartum* depression through only women’s reproductive traits, but not other pathways; 3) SNPs are independent of other risk factors and confounders. MVMR involved 3 core assumptions (Burgess and Thompson, 2015). 1) SNPs are associated with one or more women’s reproductive traits; 2) SNPs are conditionally independent of the PPD given the risk factors and confounders; 3) SNPs are not associated with a confounder of any of the risk factor-PPD associations.

### 2.2 Data sources

This analysis was obtained from the publicly available summary information of GWASs, all of which were for European populations (Table 1). The UK Biobank is a large-scale biomedical database and research resource, containing in-depth genetic and health information from half a million UK participants. The Within Family Consortium has conducted a within-family GWAS using data from 159,701 siblings from 17 cohorts to generate population (between-family) and within-sibship (within-family) estimates of per-locus genetic associations for 25 phenotypes. FinnGen aims to collect and analyze genotype-phenotype correlations in Finnish participants.

**TABLE 1:** Women’s reproductive traits and Postpartum depression datasets.

Traits	Consortium	Ancestry	Gender	Sample size	n SNPs	PMID or web link
AFS	United Kingdom Biobank	European	Female	397,338	16,359,424	34211149
AMC	The Within Family Consortium	European	Female	29,346	7,890,254	https://www.withinfamilyconsortium.com/
AFB	United Kingdom Biobank	European	Female	542,901	9,702,772	34211149
AMP	The Within Family Consortium	European	Female	11,859	6,972,488	https://www.withinfamilyconsortium.com/
LNSP	United Kingdom Biobank	European	Female	378,882	9,851,867	https://gwas.mrcieu.ac.uk/
ALLB	United Kingdom Biobank	European	Female	170,248	9,851,867	https://gwas.mrcieu.ac.uk/
NLB	United Kingdom Biobank	European	Female	180,952	10,894,596	https://gwas.mrcieu.ac.uk/
Number of stillbirths	United Kingdom Biobank	European	Female	78,879	9,851,867	https://gwas.mrcieu.ac.uk/
Number of spontaneous miscarriages	United Kingdom Biobank	European	Female	78,700	9,851,867	https://gwas.mrcieu.ac.uk/
PPD	FinnGen	European	Female	249,835	20,166,012	https://r8.finngen.fi/pheno/O15_POSTPART_DEPR

### 2.3 Data information

The accessible summary data on women’s reproductive traits was obtained from a publicly accessible database that exclusively included Europeans. (IEU OPEN GWAS PROJECT: https://gwas.mrcieu.ac.uk/, Accessed 24 July 2023). The GWAS dataset for PPD was generated from the FinnGen R8 publicly accessible data (https://www.finngen.fi, Accessed 24 July 2023) (Table 1). PPD is defined as “mental and behavioral disorders associated with the puerperium, not elsewhere classified code category” according to the International Statistical Classification of Diseases (10th revisions). No further ethical review form was necessary for this study since all participants signed informed consent forms and were found to be compliant with local ethical standards when they enrolled.

### 2.4 Selection of genetic instrumental variables

SNPs associated with women’s reproductive traits (age at first sexual intercourse, age at menarche, age at first birth, age at menopause, age at last live birth, number of live births, and lifetime number of sexual partners) were identified from the corresponding genome-wide association studies to achieve genome-wide significance (*p* < 5 × 10^−8^) and independence (linkage disequilibrium *>* 10,000 kb and r^2^ < 0.001 region based on the European sample of 1000 Genomes data) (Li et al., 2022), which to fulfill assumptions of relevance and independence. We excluded risk factors associated with *postpartum* depression (antenatal anxiety and depression, violence, abuse, obesity, and pregnancy complications.) to ensure that hypothesis exclusion was established. Proxy SNPs were not used in this study and palindromic SNPs with intermediate allele frequencies were deleted. With the PhenoScanner (http://www.phenoscanner.medschl.cam.ac.uk/), confounders linked to the result were detected and manually deleted. SNPs with F-values larger than 10 were chosen to confirm the validity of the exposure (Sanderson et al., 2019). The genetic tool was set to *p* < 5 × 10^−6^ since there were few SNPs in the exposures (number of stillbirths, number of spontaneous miscarriages) that were linked with PDD at the *p* < 5 × 10^−8^ level (Huang et al., 2021).

### 2.5 Statistical analysis

The UVMR method was used to evaluate overall causal effects. For UVMR analysis, the Inverse Variance Weighted (IVW) method was employed as the primary methodology for causative inference. The IVW method considers the effect estimates and variances (or standard errors) of each IV as weights, which are then summed to obtain a definite and accurate causal effect estimate. The MR-Egger, weighted median, and weighted mode methods were also performed as supplementary analyses to further highlight the stability and directionality of the results. Cochran’s Q statistic for heterogeneity testing. Horizontal pleiotropy tests included MR Pleiotropy Residual Sum and Outlier (MR-PRESSO) and MR-Egger regression. Whether the MR-Egger intercept was different from zero served as a test for horizontal pleiotropy. The MR-PRESSO test was performed to look for potential outliers, and adjustments were determined by removing outlier SNPs. The leave-one-out method was used to analyze the impact of individual SNP on the overall estimate of robustness. The Steiger filtering test was utilized to prevent reverse causality.

The MVMR method was chosen to evaluate straight causal effects. The IVW method was utilized as the primary analytical strategy for MVMR, with MR-Egger and weighted median as supports. Cochran’s Q statistic for heterogeneity testing. Horizontal pleiotropy tests by MR-Egger regression. We employed LASSO regression after the fact to avoid the problem of covariance across numerous exposures.

The Bonferroni adjustment (0.05/9 ≈ 0.005) was used to define significant evidence in the UVMR analysis, with results at *p* < 0.005 reflecting significant evidence and 0.005 < *p* < 0.05 reflecting suggestive evidence. Analyses were conducted using the TwoSampleMR, MR-PRESSO, and Mendelian randomization package in R version 4.3.0.

## 3 Results

### 3.1 Genetics variants selection

We performed linkage disequilibrium and removed SNPs with palindromic intermediate allele frequencies, confounders, and outliers to ensure the validity and reliability of the instrumental variables. UVMR analysis was performed on 141, 6, 49, 3, 54, 5, 3, 13, and 16 SNPs in AFS, AMC, AFB, AMP, LNSP, ALLB, NLB, number of stillbirths, and number of spontaneous miscarriages, respectively (Supplementary Tables S1-S9). The average F-values varied from 20.92 to 143.76, indicating that weak instrument bias was avoided (Supplementary Table S10).

### 3.2 UVMR results of women’s reproductive traits on PPD

**FIGURE 2:**
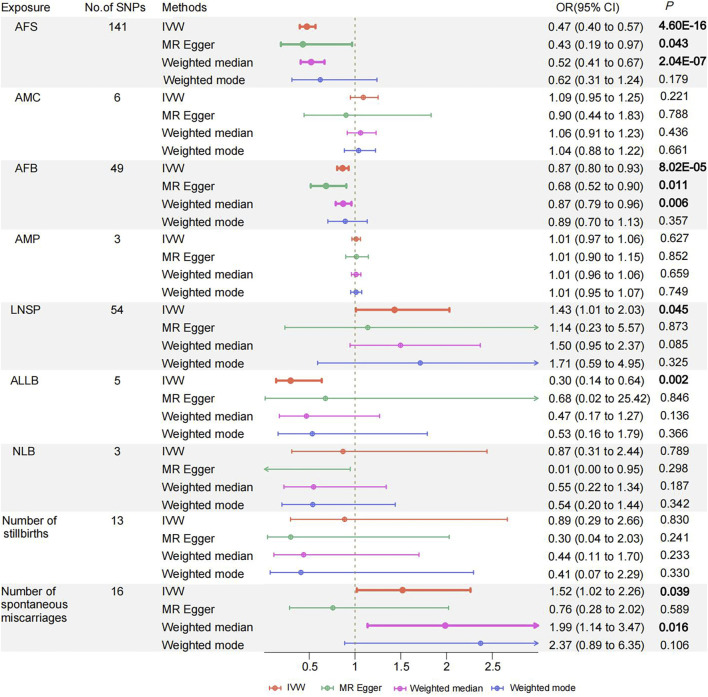
Odds ratios and 95% confidence intervals for the effect of Women's reproductive traits on Postpartum depression estimated by Univariate MR; No of SNP: number of SNPs used in MR. AFS, age at first sexual intercourse, AMC, age at menarche, AFB, age at first birth; AMP, age at menopause, LNSP, lifetime number of sexual partners; ALLB, age at last live birth; NLB, number of live births; PPD, Postpartum depression. Colors indicate for four appr oaches used in MR analyses.

The IVW method was primarily employed in the UVMR analysis to evaluate causal effects. The results showed a significant inverse causal relationship between AFS, AFB, ALLB, and PPD while supporting a suggestive direct causative association between LNSP and the Number of spontaneous miscarriages and PPD. Considering the best causal estimation, per additional year of AFS, AFB, and ALLB, the significant incidence of PPD decreases by 52.6% [95% confidence interval (CI) 0.396–0.567; *p* = 4.6 × 10^−16^], 13.5% (95% CI 0.805–0.930; *p* = 8.02 × 10^−5^), and 70.4% (95% CI 0.138–0.636; *p* = 0.002) (Figure 2; Supplementary Table S12). The odds of suggesting PPD increased by 43.1% (95% CI 1.009–2.031; *p* = 0.045) and by 51.9% (95% CI 1.021–2.262; *p* = 0.039) with each additional LNSP and number of spontaneous miscarriages, respectively (Figure 2; Supplementary Table S11). However, we did not find a causal association between AMC, AMP, NLB, and the number of stillbirths on PPD (all *p* < 0.05). The weighted median method was advised (Nazarzadeh et al., 2020) because Cochran’s Q test revealed heterogeneity in the AFS results but did not reveal horizontal pleiotropy (Q = 173.73; *p* = 0.028; Supplementary Table S12). The AFS results similarly demonstrated significance in the weighted median method (OR = 0.521, 95% CI 0.407–0.666; *p* = 2.04 × 10^−7^) (Supplementary Table S11). MR PRESSO removed one outlier (RSS = 176.267, *p* = 0.039), and causal comparisons were significant both before and after removal (before OR = 0.468, 95% CI = 0.388–0.565, *p* = 2.06 × 10–15). The results of the remaining women’s reproductive traits were tested for heterogeneity by Cochran’s Q statistic and horizontal pleiotropy by the MR-Egger intercept test (Supplementary Table S12). The leave-one-out method revealed that the IVW analyses performed by successively removing individual SNPs were typically consistent with the IVW analyses that included all SNPs, suggesting that no individual SNP had a significant effect on the results. The Steiger filtering test was successful for all exposure data.

### 3.3 MVMR results of women’s reproductive traits on PPD

**FIGURE 3:**
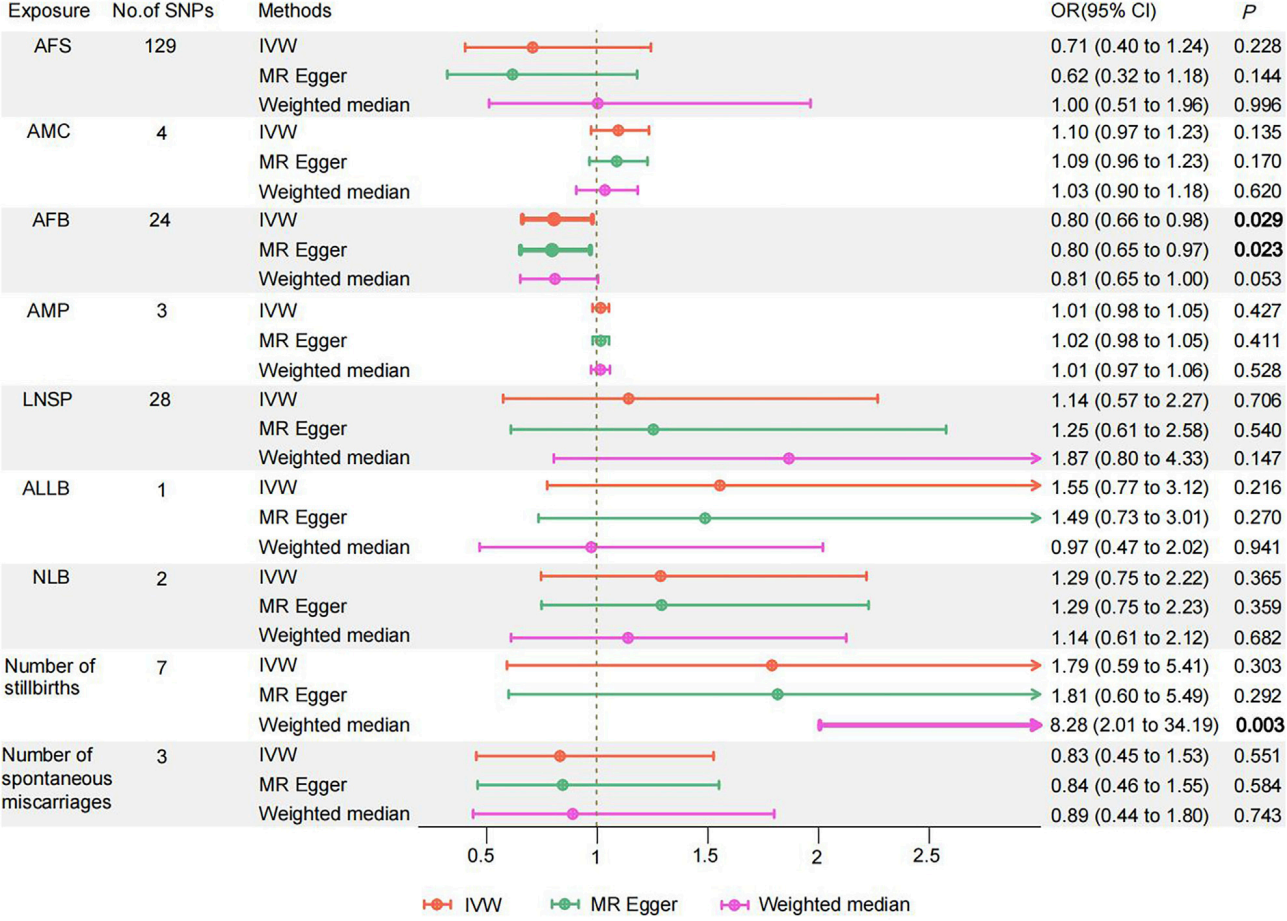
Odds ratios and 95% confidence intervals for the effect of Women' s reproductive traits on Postpartum depression estimated by Multivariate MR; No of SNP: number of SNPs used in MR. AFS, age at first sexual intercourse; AMC, age at menarche; AFB, age at first birth; AMP, age at menopause; LNSP, lifetime number of sexual partners; ALLB, age at last live birth; NLB, number of live births; PPD, Postpartum depression. Colors indicate for three approaches used in MR analyses.

MVMR analysis revealed that AFB had a direct inverse influence on the causal association of women’s reproductive traits with PPD (OR = 0.804, 95% CI 0.661–0.978; *p* = 0.029) (Figure 3; Supplementary Table S14). There was not enough evidence of a causal association for PPD and other women’s reproductive traits in the MVMR (all *p* > 0.05). The LASSO regression provided powerful support between the results obtained from the MVMR analysis, specifically for AFB (Supplementary Table S15).

## 4 Discussion

To the best of our knowledge, this is the first study to investigate the genetic causal correlations of women’s reproductive traits with PPD using MR methods, providing insights into preventing and detecting PPD by modifying one or more reproductive traits. The risk correlations between the other reproductive features and PPD were almost entirely removed after adjusting for the effects of AFB. On the other hand, after controlling for the other factors, there was a robust association between the risk of AFB for PPD. Our study identified AFB as a major risk factors for the development of PPD among women’s reproductive characteristics. Specifically, younger AFB was strongly inversely linearly associated with PPD. Our study is therefore fundamental as an attempt to fill gaps in our understanding of PPD between either causally or remotely involved women’s reproductive traits and the relevant interactions in PPD in a genomic context.

The results of UVMR analysis indicate a substantial causal association between AFS, AFB, and PPD. The study found a negative association between AFB and depression during pregnancy (McMahon et al., 2011; McMahon et al., 2007). A study comparing the psychological resilience of older (maternal age ≥38 years) and younger (maternal age <35 years) pregnant women revealed that older pregnant women exhibited greater psychological resilience (McMahon et al., 2007). At the univariate level, the older the maternal age, the lower the symptoms of depression, and the greater the psychological resistance (McMahon et al., 2007). While adolescent mothers displayed a higher likelihood of experiencing psychological problems after delivery (Aitken et al., 2016). Another study found that increasing age at first birth increased the risk of adverse pregnancy and birth outcomes, which included some risk factors for PPD such as stillbirth, death after multiple pregnancies, gestational diabetes, etc (Schummers et al., 2018). Nevertheless, multiple research has supported the claim that advanced AFB is connected with increased psychological resilience and a lower risk of PPD (McMahon et al., 2011; McMahon et al., 2007). This finding supports our hypothesis that young age at AFB is a significant risk factor for PPD. During puberty, increased hormone production causes substantial physical, psychological, and sexual behavioral changes (Lara and Abdo, 2016). Early sexual experience is generally accompanied by unsafe sexual conduct, which can lead to psychological difficulties, and the use of contraception is less frequent in this context (Greer, 1982), leading to the emergence of premature AFB. Early AFB could reflect an unorganized transition from adolescence to adulthood (Mirowsky and Ross, 2002), and younger or even adolescent primiparous women are more likely to experience PPD or to have an impact on their health and quality of life in the coming decades than primiparous women over 25 years of age (Lu et al., 2023). Another Mendelian study supported the causal relationship between AFS LNSP and MDD. However, a social study discovered that the time of a sexual experience did not significantly affect depression levels, which discovered that earlier sexual experience decreased cortisol stress levels in demanding circumstances (Brody, 2002). Our results explain the conflicting findings of the two studies mentioned.

After we included more reproductive traits in the MVMR model for adjustment, we discovered that AFB was a major risk factor for PPD and that AFS and LNSP may have produced risk associations for PPD thanks to AFB. In other words, AFB mediates the adverse implications of early sexual experience and an excessive number of sexual partners in PPD. Although educating teens about sexual conduct is beneficial in preventing PPD and other illnesses, preventing premature AFB has a greater impact on the health of mothers and offspring to the extent of PPD. The UVMR results also showed an association between ALLB, the number of spontaneous miscarriages, and the risk of PPD, but these associations were removed in the subsequent MVMR analyses. Our findings indicate that AFB may be a contributor to their association with PPD risk. The UVMR analysis typically measures the total causal association of the risk factor with the phenotype, even though one risk factor may mediate the causal effect of another risk factor. However, MVMR analysis avoids indirect causal effects and assumes that one risk factor can mediate the effect of an outcome independently of other risk factors (Burgess et al., 2014). The study confirms genetically that AFB is an independent risk factor for PPD and that premature AFB has a direct causal association with the development of PPD. However, this study was unable to ascertain whether there is a correlation between age at first birth and PPD. Comorbidities are typically the consequence of a complex interplay between genetic and environmental factors. To ensure the precision of the Mendelian study, we excluded potential environmental risk factors, which require further investigation in future studies. The effects of AFS, ALLB, LNSP, and the number of spontaneous miscarriages on PPD may be related to AFB (as shown in Figure 1). In other words, without changes in AFB, changes in AFS, ALLB, LNSP, and the number of spontaneous miscarriages may not lead to a change in PPD risk.

Childbirth is considered a major trigger for PPD, and the drop in reproductive hormones that occurs during childbirth may increase susceptibility to PPD (Meltzer-Brody et al., 2018). However, we have no proof that NLB has a hereditary influence on PPD, which is in line with the findings of certain investigations (Lynch and Prasad, 2014). On the contrary, depression negatively affects NLB (Sejbaek et al., 2013). Additional studies are required to explore the potential causal relationship going both ways. Stillbirth increases the risk of PPD up to thrice when compared to live birth (Hirtz et al., 2022). Our study indicates that AMC, AMP, and PPD are not genetically causally related, whereas previous studies have suggested that both are associated with an increased risk of depression (Wang et al., 2023). It might be a result of the genetic variations between PPD and MDD. Reproductive traits have varying causal effects on PPD and MDD, suggesting that PPD and MDD differ at the level of genetic inheritance. These two types of depression may have distinct biological processes that require further investigation.

## 5 Strengths and limitations

This study has several strengths. Firstly, Two-sample Mendelian randomization using large GWAS sample sizes for both exposures and outcomes is considerably precise and accurate, filling the observational gaps and weaknesses in the studies on PPD. Secondly, a crucial tenet of UVMR and MVMR is that exposure only impacts outcomes via this pathway. This presumption reduces the confounding of risk variables and confounders in observational research to a greater extent. Thirdly, The assessment of the causal connection between each of the modifiable exposures and the risk of PPD is made possible by the use of multiple genetic variants as tools for each of the women’s reproductive traits. As a result, we were able to successfully perform sensitivity analysis to identify and account for directional pleiotropy. Fourthly, this study focused on women’s reproductive traits, covering multiple aspects of the traits and providing a comprehensive analysis of possible influences on *postpartum* depression. Finally, the cases included in this study were from European populations, which avoids genetic heterogeneity in the population to the greatest extent possible and ensures the accuracy of the results.

This study also has several limitations. Firstly, the limited selection of SNPs with partial exposure (number of stillbirths, number of spontaneous miscarriages) used as a genetic instrument forced us to choose more lenient screening conditions (*p* < 5 × 10^−6^), which may have reduced the validity of the results. However, the direction of the GWAS effect was consistent across significant studies, the analysis when combined with individual SNPs was still valid, and the genetic instrument did not exhibit weak instrumental bias. Consequentially, our results are accurate, and the number of corresponding SNPs can be increased in the future by gathering more comprehensive GWAS data. Secondly, there was heterogeneity in the AFS study’s findings, which might be ascribed to possible bias caused by the existence of potential unrecognized confounders. However, our results satisfied the directional pleiotropy requirements of the MR-Egger and MR-PRESSO tests and also demonstrated relevance when using the weighted median method. The results are robust and reliable after the final elimination of potentially important SNPs using the leave-one-out method. Thirdly, The scope of this study was limited to European populations, therefore the results may not be applicable to other races, and the same type of study could be conducted in other races for comparison in the future. Finally, the privacy restrictions imposed by the UK Biobank meant that we were unable to collect a more precise age-sex breakdown and thus definitively prevent the optimal age of first birth for PPD. Future studies should collect more data on male reproductive traits and precise age nodes in order to more precisely characterize their respective effects on PPD. Notably, this study offers the closest RCT on women’s reproductive traits and the risk of *postpartum* depression despite these potential drawbacks.

## 6 Conclusion

This study extensively investigated the genetic underpinnings of women’s reproductive traits and PPD to assess their causal relationships. Genetic evidence confirms that AFB has a significant direct causal effect on PPD. It is possible to greatly reduce the risk of PPD by adjusting modifiable reproductive traits, which facilitates the development of deeper and more individualized studies of biological mechanisms.

## Data Availability

The original contributions presented in the study are included in the article/Supplementary Material, further inquiries can be directed to the corresponding authors.
