# Supplementary material for: Causal relationship between Women’s reproductive traits and postpartum depression: a multivariate mendelian randomization analysis
Source: Front Genet. 2024 Oct 11;15:1434762. doi: 10.3389/fgene.2024.1434762 (PMC11502326; doi:10.3389/fgene.2024.1434762)
Supplement: Supplementary file 1 [file DataSheet1.PDF]

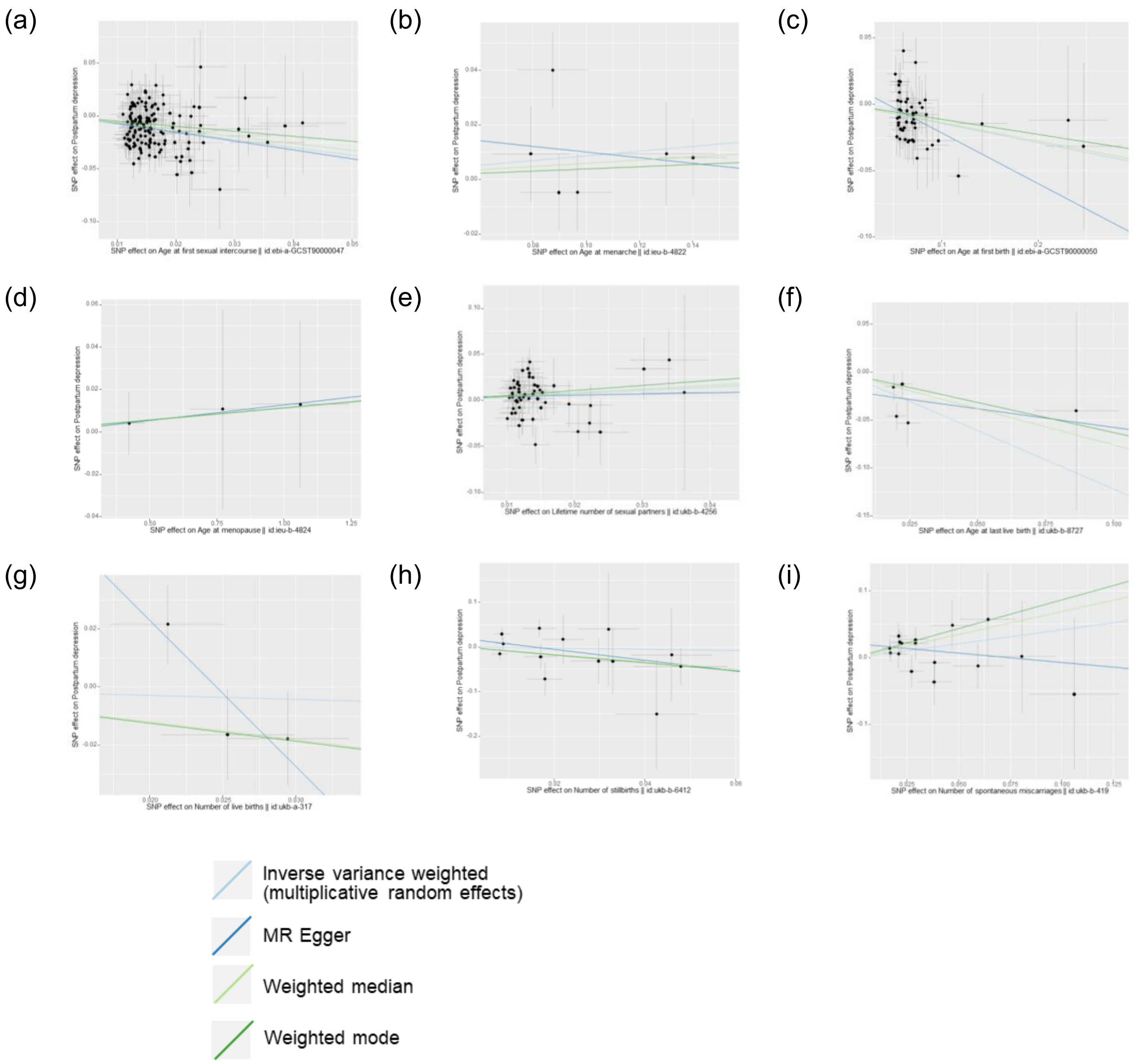

Supplementary figures 1. Scatter plots for effect sizes of SNPs for women's reproductive traits and Postpartum depression. (a) Funnel plots for age at first sexual intercourse; (b) Funnel plots for age at menarche; (c) Funnel plots for age at first birth; (d) Funnel plots for age at menopause; (e) Funnel plots for lifetime number of sexual partners; (f) Funnel plots for age at last live birth; (g) Funnel plots for number of live births; (h) Funnel plots for number of stillbirths; (i) Funnel plots for Number of spontaneous miscarriages. The x-axis represents the MR estimate of individual variants; the y-axis represents the inverse of their standard error. Colors indicate for approaches used in univariable MR analyses.

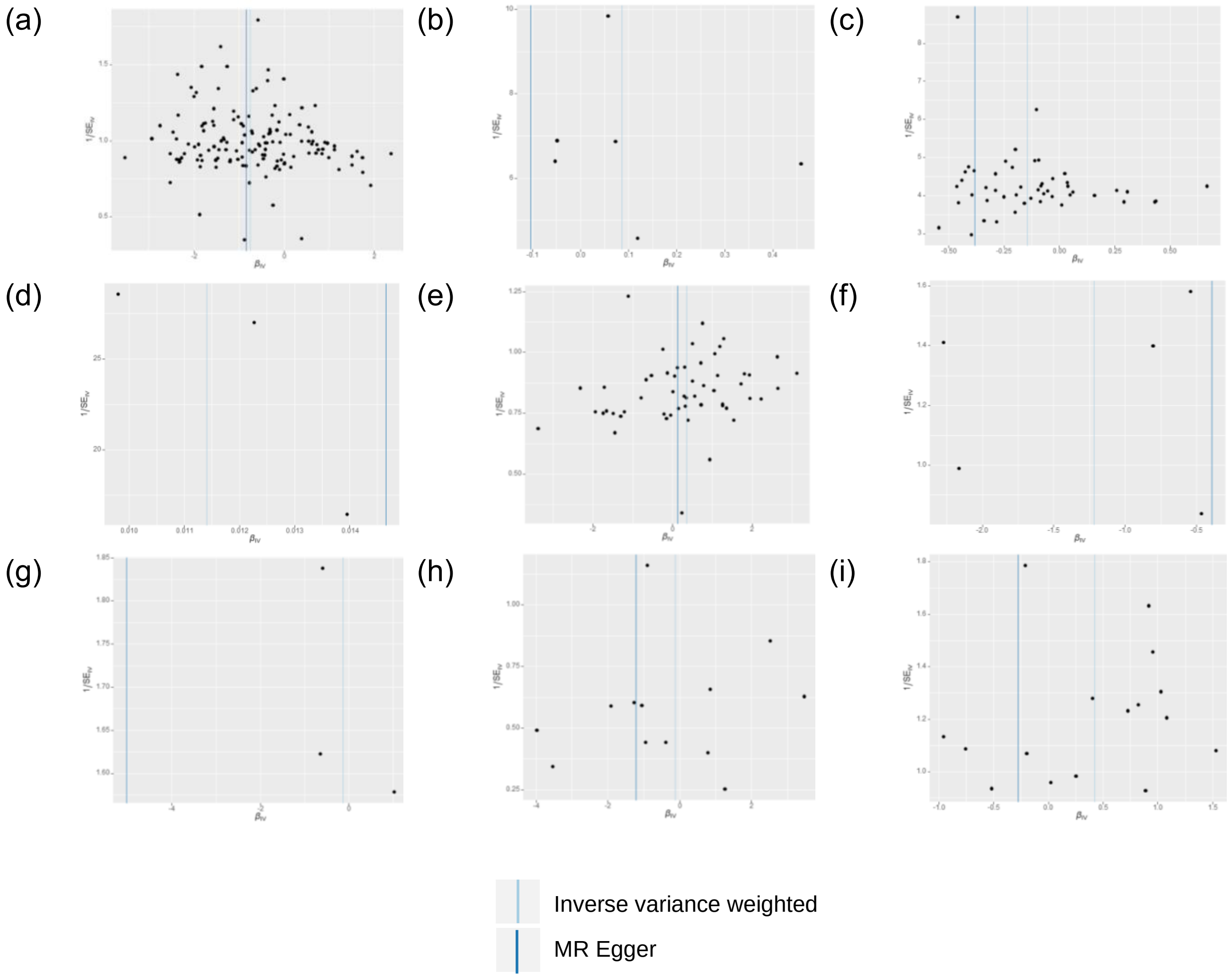

Supplementary figures 2. Funnel plots to show symmetrical distribution of individual variant estimates around the point estimate. (a) Funnel plots for age at first sexual intercourse; (b) Funnel plots for age at menarche; (c) Funnel plots for age at first birth; (d) Funnel plots for age at menopause; (e) Funnel plots for lifetime number of sexual partners; (f) Funnel plots for age at last live birth; (g) Funnel plots for number of live births; (h) Funnel plots for number of stillbirths; (i) Funnel plots for Number of spontaneous miscarriages. The x-axis represents the MR estimate of individual variants; the y-axis represents the inverse of their standard error. Colors indicate for approaches used in univariable MR analyses.
